# Development of Chocolates with Improved Lipid Profile by Replacing Cocoa Butter with an Oleogel

**DOI:** 10.3390/gels7040220

**Published:** 2021-11-18

**Authors:** María Dolores Alvarez, Susana Cofrades, María Espert, Teresa Sanz, Ana Salvador

**Affiliations:** 1 Instituto de Ciencia y Tecnología de Alimentos y Nutrición (ICTAN-CSIC), 28040 Madrid, Spain; scofrades@ictan.csic.es; 2 Instituto de Agroquímica y Tecnología de Alimentos (IATA-CSIC), 46980 Valencia, Spain; mespert@iata.csic.es (M.E.); tesanz@iata.csic.es (T.S.); asalvador@iata.csic.es (A.S.)

**Keywords:** foam-templated approach, edible oleogel, hydroxypropyl methylcellulose, cocoa butter, chocolate, lipid profile, Casson model, sensory analysis

## Abstract

The reformulation of chocolates seeks to find innovative alternatives to cocoa butter (CB) that are more economical and adhere to nutritional recommendations to replace saturated fats with unsaturated ones. In this research, chocolates were elaborated by substituting CB with an oleogel (OG) formulated with hydroxypropyl methylcellulose (HPMC) as an entrapper of sunflower oil by using the foam-templated approach. Four different CB/OG blends were prepared and characterized as potential CB substitutes (100/0 control), at replacement levels of 30%, 50%, 70% and 100% (70/30, 50/50, 30/70 and 0/100 blends), and subsequently, CB/OG-based chocolates (CB/OG-Ch) were formulated (100/0-Ch, 70/30-Ch, 50/50-Ch, 30/70-Ch and 0/100-Ch). Both the CB/OG blends and the CB/OG-Ch counterparts were characterized by dynamic and stationary rheology, hardness, thermal parameters, microstructure, and oil-binding capacity; in addition, the lipid profile of the chocolates was analyzed, and a sensory analysis was performed. Increasing the OG proportion in the CB/OG blend weakens the rigidity and strength of the fat-crystal network conferred by the CB, and decreases both its viscoelasticity and thermal parameters, but the differences between all the different properties and parameters of the CB/OG-Ch samples diminished in presence of the other ingredients used in the chocolate formulation. Sensory analysis evidenced that it is possible to replace up to 70% of CB with the OG, although from a technological point of view a replacement level of 50% would seem more appropriate. As compared to 100/0-Ch, 50/50-Ch and 30/70-Ch involve saturated fat reductions of 55% and 37%, respectively.

## 1. Introduction

Chocolate is primarily composed of its natural fat, i.e., cocoa butter (CB) [1], with a total solid content of 65–75% [2]. From a colloidal perspective, chocolate comprises micro particles (e.g., sugar, cocoa powder and milk powder) dispersed within the CB continuous phase [3,4], although other fats from milk and vegetables can be present, depending on specific formulations. In chocolate, the continuous phase provides a network structure that maintains the spatial position of the dispersed phase, determining its microstructure, texture, crystallization, and rheological properties, and conferring chocolate its characteristic flavor [5]. Palmitic (P; C16:0), stearic (S; C18:0) and oleic (O; C18:1) acids are the three major fatty acids (FA) present in CB. When these FA are linked through a glycerol backbone, they form triglycerides (TAG) such as POS, SOS, and POP at 28.2%, 35.0% and 32.5%, respectively [1,6,7]. This specific range of TAG species composition contributes to the unique physical properties of CB, including its melting temperature, crystallization and polymorphisms [8], and its appealing sensory characteristics.

Due to the rising trend in the consumption of cocoa products, the demand for CB is expected to grow in the next years [9]. However, on the one hand, CB production and supply are limited due to poor yield returns, variation in crop yield and labor shortage [5,10,11], which implies that CB is more expensive than other vegetable fats and oils. On the other hand, saturated fat in CB is close to 60%, and therefore, the consumption of chocolate confectioneries increases the risk of cardiovascular disease and induces insulin resistance [12]. To address supply shortage, CB is replaced by other cheaper vegetable fats and oils, and the food manufacturing industry has mainly leaned towards CB alternatives (CBA), grouped into CB substitutes (CBS), CB replacers (CBR), and CB equivalents (CBE) depending on either their functionality or similarity to CB [7,8,10]. However, only six vegetable fats are permitted to be used as a CBE according to the EU Directive 2000/36/EC [13], and in real chocolate products, the addition of vegetable fats other than CB, is limited to a maximum of 5% [7]. In addition, CBEs are produced with a TAG profile resembling that of CB. Therefore, they do not contribute to the needed improvement of the lipid profile of chocolate finished products.

In this context, oleogels have been used in the formulation of chocolates and chocolate-derived products to decrease saturated fatty acid (SFA) contents, as well as to increase the melting temperature [12]. In a product like chocolate, oleogels present interesting advantages once they can provide the desired thermal stability [14]. Aiming at producing heat-resistant chocolate and controlling oil migration, CB has been partially replaced by ethyl cellulose, waxes and mono glyceride oleogels [15,16,17,18,19]. Additionally, shellac oleogels have been explored as structuring agents in chocolate, spreads and cakes [20,21].

Nevertheless, using polymeric-based oleogelators, including different sources of proteins [22,23,24] and polysaccharides like celluloses ethers [25,26,27,28,29,30,31,32], has attracted a great deal of research interest. These oleogels are obtained by indirect approaches to oil structuring, due to the insolubility of these biopolymers in oil, with the advantage of widening substantially the types of oleogelators that can be used [14].

Between the indirect methodologies that are being used [33], most of the early work on oleogels has focused on the emulsion-templated approach [22,26,29,31,32,34], which involves the preparation of an emulsion stabilized by a hydrophilic gelator, followed by water removal [35], usually through conventional drying in an oven. Hydroxypropyl methylcellulose (HPMC) is used as an oleogelator for practical applications in structuring different confectionary products, including chocolate spreads [36] and bakery creams [37]. However, the elevated temperatures of conventional drying and the long drying times used can be detrimental to the quality of the oil, causing oleogels to present a low oxidative stability [33,34]. A promising alternative to conventional drying is freeze drying. In this context, Patel et al. [25] introduced the foam-templated approach to create a porous polymeric structure with excellent oil sorption properties. Despite the simplicity of preparation without the use of high temperatures, this approach is still underexplored, and it has only been tested for practical applications in the structuring of sandwich cookie creams [27], peanut butter [28], muffins [30], and cakes [24]. Therefore, this foam-templated approach should generate interest among researchers since it could help to expand practical applications of oleogels in healthier food products. In this context, an optimization of the freeze-drying cycle in the formulation and process development is to minimize the cost by energy consumption.

To the best of our knowledge, oleogels obtained by the foam-templated approach have not been used to replace CB in chocolate. Herein, we chose sunflower oil as representative of a vegetable oil rich in monounsaturated fatty acids (MUFA) to prepare a sunflower oil-based oleogel (OG) from water-soluble HPMC by using the foam-templated approach, as the continuous phase in healthier chocolate with a partial (30–70%) or total (100%) replacement of CB. The aim of the present study was to explore the effect of this OG, as a substitute for CB, on physicochemical properties (such as rheological and thermal behaviors, texture, oil-binding capacity) of both the CB/OG blends and the chocolate counterparts (CB/OG-Ch). The sensory properties and the lipid profile of chocolates were also investigated.

## 2. Results and Discussion

### 2.1. Lipid Profile of CB/OG-Ch Samples

All CB/OG-Ch were formulated so as to have a similar amount of fat, as may be observed in Table S1, which shows the theoretical nutritional composition of each chocolate. In turn, the effect of the percentage replacement of CB with the OG on the fatty acid (FA) profile of the five CB/OG-Ch samples is shown in Table 1. Predominant FA contents detected in the 100/0-Ch control were stearic, oleic, palmitic, linoleic and arachidic acids, in this decreasing order, confirming previous findings in either CB [38] or in dark chocolate [19,39]. This represents SFA and unsaturated fatty acid (UFA) compositions of approximately 18 and 11%, respectively. However, oleic and linoleic acids were the major FAs in 0/100-Ch without CB, attributable to the presence of high oleic refined sunflower oil (HORSO), which is characterized by an elevated quantity of MUFA (>80%), mainly oleic acid [6].

In addition, increasing the OG proportion in the CB/OG blend involves a significant (*p* < 0.05) reduction in the SFA content and a significant increase in both MUFA and PUFA contents, which is consistent with an improved lipid profile. As compared with the 100/0-Ch control, 50/50-Ch and 0/100-Ch samples involve 37 and 80% saturated fat reductions, respectively. Improvements in the lipid profile are associated with the significant reduction of palmitic acid, which is related to some chronic degenerative diseases (cardiovascular disease, some types of cancer, etc.), as well as the significant increase in oleic acid, which has several known beneficial effects, particularly on cardiovascular health [40]. On the other hand, partial and total replacement of CB with the OG in the CB/OG blend leads to a significant increase in the PUFA/SFA ratio of the CB/OG-Ch counterparts (Table 1), being closer to the recommended values (>0.4) [41]. As well, AI and TI indexes provide an indication on the nutritional quality of lipids, with low values representing healthier foods, having better nutritional quality of FA [42]. Both AI and TI indexes decreased as the percentage of substitution of CB with the OG increased, and were significantly lower in 0/100-Ch, suggesting a high cardioprotective effect.

### 2.2. Rheological Properties of CB/OG Blends and CB/OG-Ch Counterparts

#### 2.2.1. Amplitude Sweep Tests

**Figure 1:**
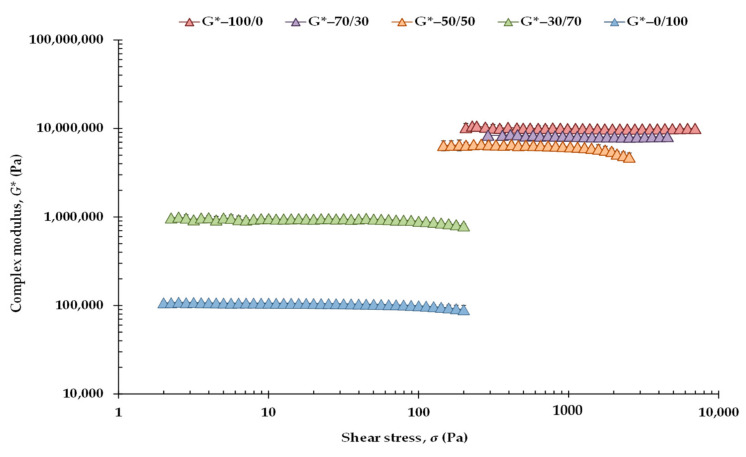
Complex modulus (G*) as a function of the applied shear stress at 1 Hz and at 20 °C for the CB/OG blends. Shear stress ranged from 2 to 10,000 Pa depending on the CB/OG blend measured.

Amplitude sweeps were initially run to determine the linear viscoelastic (LVE) range at 20 °C of both the CB/OG blends (Figure 1) and the CB/OG-Ch counterparts (Figure S1). The G* values corresponding to the control blend (100/0) were higher than those of the rest of the blends, even with an order of magnitude of up to two logarithmic decades above the values corresponding to the OG without CB (0/100). Critical (maximum) values of shear stress (*σ*_max_) and shear strain (*γ*_max_), at which G* values begin to show a significant deviation from their previously constant values were calculated in accordance with Solo-de Záldivar et al. [43], i.e., with a tolerable deviation with respect to G* initial value of 10%. Therefore, G*_max_ refers to critical values of G* on the limit of LVE range.

Table 2 shows the effect of the percentage of CB substitution on the rheological parameters limiting the LVE range for the CB/OG blends. For each CB/OG blend studied, the stress within the LVE range chosen to run subsequent small amplitude oscillation sweeps (SAOS) tests was also valid for each CB/OG-Ch counterpart (data not shown). Significant differences can be observed between these rheological parameters corresponding to the CB/OG blends. *σ*_max_ provides information related to the structural and physical stability (density) of the formed network, *γ*_max_ is a measure of its conformational flexibility, and G*_max_ is a measure of the total resistance of the sample to applied deformation, representing the rigidity of the network [43]. The 100/0 control showed the highest *σ*_max_ and G*_max_ values, and therefore, the densest and most rigid fat-crystal network, compared with the CB/OG blends which contained OG in higher or lower proportions.

As compared to the 100/0 control, the 0/100 sample without CB exhibited a much narrower LVE range, with a *σ*_max_ of 142 Pa, although this value is higher than that previously observed by Patel et al. [25] in an OG obtained under comparable conditions. These authors monitored a *G*’ value at the LVE range on an order of magnitude of 10^4^, and a crossover point of *G*” and *G*’ at 130 Pa. However, in this OG, and even above 200 Pa, the phase angle (*δ*) was close to 7°, and the *G*ʹ value (practically identical to the G* value) at the LVE range was in order of magnitude 10^5^ (Figure 1), thus indicating stronger internal forces and higher gel stiffness than in similar oleogels previously obtained.

In addition, the highest conformational flexibility corresponded to the polymeric network formed by the OG in the absence of CB, with a much higher *γ*_max_ value than that corresponding to the rest of the CB-containing blends (Table 2). This result was expected as the SFA content of the OG was much lower than that of mixtures containing CB (Table 1), which is also associated with the formation of a different three-dimensional network microstructure in the 0/100 sample [44]. It is also well known that CB confers on chocolate the proper ability to break easily [4], which it is directly related to a low conformational flexibility. Besides, both 100/0 and 70/30 mixtures had the lowest tan *δ*, i.e., the strongest internal network structures and the highest viscoelasticity. However, the 0/100 sample also had a very low loss factor (around 0.1), confirming previous findings in other oleogels made with sunflower oil, HPMC and xanthan gum using the emulsion-templated approach [32].

#### 2.2.2. Frequency Sweep Tests

Viscoelastic moduli as a function of frequency at 20 °C and within LVE range are represented in Figure S2 for both the CB/OG blends and the CB/OG-Ch counterparts. In both matrix types, there was a similar trend in dynamic rheological behavior, although the relative ranking of viscoelastic data was very different. However, the addition of sugar, lecithin, cocoa powder and milk powder into the corresponding CB/OG blend for obtaining the chocolates reduced noticeably the differences in the rheological behavior of the 100/0-Ch, 70/30-Ch and 50/50-Ch chocolates, as compared with the same single CB/OG counterpart blends. In all CB/OG mixtures and in the homologous chocolates, the *G*ʹ value is higher than that of *G*ʺ across the entire frequency range tested. However, there are very significant differences between the values of *G*ʹ and *G*ʺ in the different samples, which are clearly dependent on the percentage of replacement of CB with the OG in each blend and chocolate. Nevertheless, in both matrices, the differences between the elastic and viscous moduli decrease as the percentage of CB replacement increases.

On the other hand, the frequency dependence of the viscoelastic moduli also differed significantly in the different CB/OG matrices. For instance, in the 100/0 and 100/0-Ch control samples, the values of *G*’ and *G*” increased and decreased when frequency was incremented in the range studied by 1 and 9%, and by 3 and 9%, respectively. However, in the absence of CB (0/100 and 0/100-Ch matrices), the values of *G*’ and *G*” increased with the applied frequency by 28 and 44%, and by 28 and 6%, respectively. Therefore, in the CB/OG-Ch chocolates, the frequency dependence of *G*’ also increased as CB decreased in the CB/OG mixture. Nonetheless, the frequency dependence of *G*” was much lower in 0/100-Ch than in 100/0-Ch due to the presence of other ingredients.

The observed differences allow classifying the structure of the studied systems in terms of different types of gels. Both the 100/0 and 100/0-Ch matrices showed strong or true gel-like behavior, whereas 0/100-Ch and 0/100-Ch exhibited a weak gel-like behavior. However, experimental data show that all the samples exhibited solid-like behavior. In turn, Table 3 shows the *G*’, *G*” and tan *δ* values at 1 Hz frequency for both the CB/OG blends and their CB/OG-Ch counterparts. In both matrices *G*’ and *G*” values decreased as the OG content was incremented, in good agreement with the decreasing amount of crystalline CB present in the matrix, although the differences between the parameter values corresponding to the 100/0-Ch, 70/30-Ch and 50/50-Ch samples are hardly significant, reflecting the existence of quite similar internal structures. The 100/0 control exhibited a high elastic modulus (>10^7^ Pa) at 20 °C associated with a fat-crystal network exhibiting a very viscoelastic behavior at a very small deformation. *G*’ can be considered as an indicator of the macroscopic consistency of a fat-crystal network that provides information on the intact network structure [45]. These authors reported a *G*’ value at 1 Hz of ≈ 12 MPa for CB with a solid fat content of 75%, which is quite similar to the *G*’ value obtained in this study for both 100/0 and 100/0-Ch. However, few investigations have been found in the literature where the rheological properties at small deformations have been determined in either CB or chocolates. On the contrary, it is well known that CB has a unique triglyceride composition, responsible for its various polymorphic crystallized forms that determine its chemical and physical properties [46]. Additionally, its fatty acid composition determines the way in which liquid CB solidifies, which affects its final texture and microstructure [38].

With regard to the *G*’ and *G*” values obtained in the HPMC-based OG (0/100 sample), they remained around 10^5^ and 10^4^ Pa, respectively (Table 3), in the frequency range studied (Figure S2). The sunflower oil was also converted into a solid form using a foam-structured HPMC (at 4% *w*/*w*) for replacing solid fat in muffins [30]. For the same frequency range, in the HPMC oleogel without butter these authors obtained log *G*’ and log *G*” values around 5 and 4 (Pa) respectively, which are similar to the values obtained in our 0/100 sample.

#### 2.2.3. Temperature Sweep Tests

**Figure 2:**
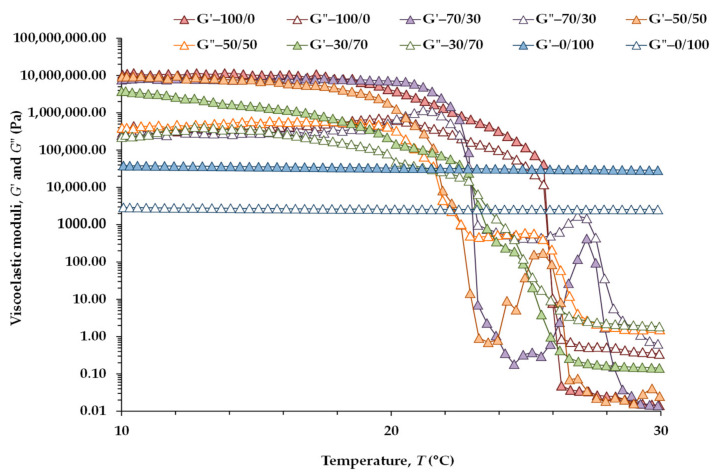
Viscoelastic moduli (*G*’: filled symbols; *G*”: open symbols) at 1 Hz as a function of the temperature for the CB/OG blends. Stress ranged from 100 to 1000 Pa depending on the CB/OG blend measured.

To analyze the effect of heating on the structure of the CB/OG blends, temperature sweeps were carried out from 10 to 40 °C (Figure 2). For this purpose, the imposed shear stresses were chosen within LVE range at the start of the heating. However, at temperatures above 20 °C, a phase change occurred in the blends containing CB, and therefore the applied stresses do not guarantee the LVE of the samples. Table 4 shows *G*’ and *G*” values at 10 and 30 °C of all five CB/OG systems together and the crossover temperature of both moduli.

The moduli values at 30 °C are expressed in Pa, in order to avoid excessively large numbers, considering that the CB/OG blends, with the exception of 0/100, are in a liquid state at this temperature. In addition, all the blends containing CB showed a liquid-like behavior between 30 and 40 °C and, therefore, the *G*’ and *G*” values at 40 °C were omitted. In turn, the viscoelastic data at 30 °C should be considered with caution, because under the applied conditions, the blends with CB are out of the LVE range.

Please note that for the CB/OG blends, the viscoelastic properties were higher at 10 °C (Table 4) than at 20 °C (Table 3), in response to a more complete crystallization of CB fat crystals at a lower temperature. Between 10 and 20 °C all the CB/OG blends exhibit a solid-like behavior, but in 100/0, 70/30, 50/50 and 30/70, this is, in all the systems with CB, between 22 and 26 °C, a crossover between *G*’ and *G*” occurs at a specific temperature (Figure 2) in response to the melting of the fat crystals present. Above this temperature, the samples exhibit a liquid-like behavior (*G*” values above *G*’). In addition, there was an increase of the viscoelastic moduli for 70/30 and 50/50 blends at temperatures close to 26–28 °C (Figure 2), reflecting that for the imposed stresses the rheological behavior of these blends is not lineal. This crossover temperature is significantly higher in the absence of the OG, due to the higher amount of saturated TGA [3] in the 100/0 control. However, there were no significant differences among the temperatures obtained for the mixtures with both ingredients. On the other hand, these crossover temperatures obtained in the four CB-containing systems fall in all cases between the peak and complete melting temperatures obtained in the CB/OG blends by differential scanning calorimetry (DSC) (Section 2.3). Therefore, this crossover temperature of the viscoelastic moduli can be considered a valid estimation of the melting point temperatures obtained by DSC as previously shown [6]. Please note that the sunflower-HPMC OG (0/100) displayed only a slight softening between 10 y 30 °C (Table 4; Figure 2). Therefore, it is possible to infer that the 0/100 sample presents very high thermal stability. In addition, Patel et al. [25] observed that in a similar OG type, *G*’ had a value of above 10^4^ Pa even at 100 °C.

In CB and sunflower oil-based cocoa butter equivalents (CBE), and using a starch pasting cell, Kadivar et al. [47] showed that SAOS experiments can also provide information on the three steps of crystallization, including primary crystallization, microstructure development of the fat-crystal network and macroscopic properties at isothermal temperature of 20 °C after being held at 70 °C for 15 min and cooled from 70 to 20 °C at 10 °C/min.

#### 2.2.4. Flow Behavior

**Figure 3:**
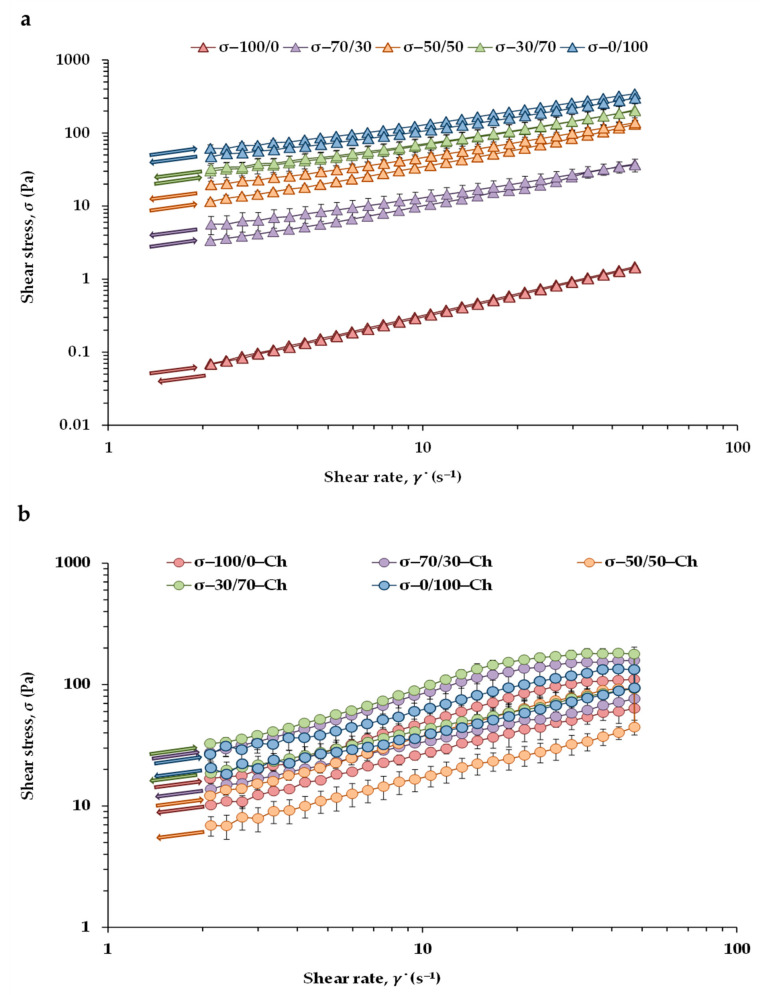
Flow curves (ramp up and ramp down) of shear stress as a function of shear rate at 40 °C: (**a**) for the CB/OG blends; (**b**) for the CB/OG-Ch chocolates.

Viscosity of CB and chocolate is directly related to product quality. The flow behavior of molten chocolate has been extensively studied using steady measurements [19,47,48,49]. The flow curves of the CB/OG blends and CB/OG-Ch counterparts obtained at 40 °C are shown in Figure 3. With the exception of the control 100/0, which showed a flow behavior close to the Newtonian one, the rest of the studied matrices behaved as non-Newtonian fluids with yield point. Moreover, in both matrices, viscosity decreases with shear rate due to a structural destruction caused by hydrodynamic forces. However, there was no direct proportionality between viscosity and shear rate in CB/OG-Ch (Figure 3b). For this reason, in the CB/OG blends, the Casson model was fitted to the shear rate-shear stress data from both upward and downward ramps, while in the CB/OG-Ch counterparts the Casson model was fitted to data only with the downward flow curves.

In addition, with the exception of pure CB, which showed a time-independent behavior, the rest of the samples exhibited a time-dependent or thixotropic behavior as evidenced by the presence of a hysteresis loop during the subsequent recovery of shear stress when the flow was discontinued. However, interesting differences were observed in the flow behavior depending on the matrix type (CB/OG blends or CB/OG-Ch). The 0/100 sample, containing only HPMC sheets dispersed throughout the oil continuous phase, showed a thixotropic behavior. In contrast, in the 30/70, 50/50 and 70/30 blends, when CB crystals were also dispersed in the oil together with cellulose ether sheets, an antithixotropic behavior was observed with stress values in descending order of shear rate being higher than those in ascending order (Figure 3a). In turn, all the CB/OG-Ch counterparts exhibited a thixotropic behavior (Figure 3b), although with less differences among them as compared with the CB/OG blends.

The results of the effect of the percentage of CB replacement with the OG on the rheological parameters obtained from the Casson model fit together with other rheological properties derived from flow curves are presented in Table 5. Casson yield stress is the stress necessary to initiate the flow, whereas plastic viscosity is related to the resistance offered to keep it [19]. In the CB/OG blends, all the steady rheological properties presented a direct relationship with the OG content. The 0/100 sample without CB displayed the highest Casson yield stress and plastic viscosity values, followed by 30/70, 50/50 and 70/30, respectively, in that order. This result reflects that in the OG without CB the continuous liquid oil phase is immobilized in a network of strong self-assembled molecules conferring a higher thermal stability as compared to that of the samples containing lower amount of OG. In addition, the lack of solid content in the 100/0 control sample at 40 °C due to the presence of the high-melting TAG [47], would justify its almost null *σ*_CA_ value. In the CB/OG blends, the trend of Casson yield stress was similar in the rest of the rheological properties calculated (Table 5); decreasing the OG content and, therefore, the amount of oil and dispersed HPMC, led to significant decreases in Casson plastic viscosity and thixotropy. Thixotropy is an indicator of the degree of particle aggregation in a suspension, and the extent to which thixotropy increased was also in accordance with the increase of the OG content in the blend, corroborating previous findings [19]. The high thixotropy observed in other oleogels has been attributed to their high sensitivity to shear, as excessive shear leads to network damage caused by a large liquid volume fraction [50], implying particle aggregation due to low interaction energy at low fat levels [51].

As compared with the differences observed between the CB/OG blends, the differences between the steady rheological parameters corresponding to each of the CB/OG-Ch counterparts decreased in the presence of other ingredients (Figure 3b, Table 5). The 100/0-Ch control showed a Casson yield stress value of 4.5 Pa, which has been attributed to interactions among dispersed sugar particles [19]. This value is also similar to that previously reported in white chocolate [49]. There were no significant differences between the thixotropy at 5 s^−1^ values of 100/0-Ch and 0/100-Ch, which could be ascribed to the presence of lecithin acting as a lubricating agent and decreasing the interaction between particles. Reductions in *σ*_CA_ have also been reported as lecithin was incremented from 0.3 to 0.5% [51]. In accordance with Kadivar et al. [52], lecithin molecules coat the sugar and cocoa particles; therefore, yield stress correspondingly decreases as more diacylglycerols are present in the chocolate.

### 2.3. Thermal Parameters of CB/OG Blends and CB/OG-Ch Counterparts

DSC was performed to analyze the effect of the percentage of CB replacement with the OG on the melting behavior of both the CB/OG blends and the chocolate counterparts. Thermograms obtained for the different CB/OG blends and CB/OG-Ch counterparts are illustrated in the Figure S3. In turn, the melting parameters of all the samples are summarized in Table 6.

The onset melting temperature (*T*_mo_) of genuine CB was significantly higher than those of all CB/OG blends containing either CB or OG. In contrast, the highest peak melting temperature (*T*_mp_) corresponded to the 70/30 sample, whereas there were no significant differences between the completion melting temperatures (*T*_mc_) of 100/0 and 70/30, with the highest CB content. The melting parameters of the CB/OG blends changed with increasing amounts of the OG. All melting temperatures of 50/50 and 30/70 blends were significantly lower than those of the 100/0 control (within 2 to 4 °C), and no thermal change in the melting curve was detected in the pure OG between 5 and 80 °C (Figure S3a). This result corroborates the high thermal stability of this OG as observed from the temperature sweeps (Figure 2). The reason for this is that no crystals are formed in this OG obtained by the foam-templated approach due to the absence of gelator molecules [3]. This is in consonance with previous studies because the incorporation of liquid oil from an OG causes changes in the polymorphic forms and melting properties [46]. In fact, in the CB/OG blends, the *ΔH*_m_ also decreased significantly as the OG content increased, which is attributed to the presence of a higher amount of liquid oil entrapped in the system.

The melting parameters of the 100/0 sample were consistent with the literature, because in CB similar endothermic onset, peak and completion temperatures at 14.0, 22.3 and 29.5 °C, respectively, has already been reported by several authors [4,19,52], as well as melting enthalpies. However, higher melting temperatures for CB have been also quantified by other authors, with *T*_mo_ and *T*_mc_ at 30.9 and at 38.6 °C, respectively [8]. This discrepancy is likely due to the presence of different proportions of polymorphic forms in the distinct CB samples analyzed. It is well known that the TGA compositions of CB are responsible for its complex polymorphic forms, namely *γ*, *α*, *β*’, *β*_V_, *β*_VI_, with increasing order of melting temperatures and ascending stability [53]. In CB, DSC studies revealed that higher values of onset and peak temperatures and enthalpy of melting were related to the polymorphic form *β*_V_ [46], and dominant peaks at 4.6 Å [8]. However, these differences could also be explained by the variations in TAG content between dissimilar CB samples, although polymorphic transitions are also possible depending on time and temperature of storage and tempering [52].

With regard to the CB/OG-Ch counterparts, and as observed for the rheological properties, the differences in the thermal behavior were smaller than in the CB/OG blends (Table 6. There was no thermal change in the thermograms corresponding to the chocolate made without CB (Figure S3b), in consonance with the results obtained for the 0/100 blend. There were no significant differences between *T*_mo_ and *T*_mp_ of all CB/OG-based chocolates, whereas the highest *T*_mc_ corresponded to the 70/30-Ch sample. However, as observed in the CB/OG blends, the *ΔH*_m_ significantly decreased as the OG content increased in chocolates due to both the lower fat content (Table S1) and higher PUFA amount (Table 1). Afoakwa et al. [2] also reported that enthalpy is reduced in products with lower fat contents. The *ΔH*_m_ values of the CB/OG-Ch samples are also lower than those corresponding to the CB/OG blends, likely due to decreased CB content in the formulated chocolates. On the other hand, the *ΔH*_m_ values of the CB/OG-Ch samples are similarly inferior to those reported by other authors in dark and white chocolates [49,53]. However, the *T*_mc_ of the 100/0-Ch control was close to those reported by Afoakwa et al. [53] in optimally tempered dark chocolates, which ranged between 32.5 and 33.6 °C. However, as previously suggested [54], the rheology and the physical stability of oleogels developed to be used as food ingredients should be tested over a wide temperature range to fully assess their functionality.

### 2.4. Textural Properties of CB/OG Blends and CB/OG-Ch Counterparts

Examples of the force–time curves obtained in both formulated CB/OG blends and CB/OG-Ch counterparts are shown in Figure S4, whereas corresponding mean textural parameter values derived are presented in Table 7. In both matrices, it is possible to appreciate a different mechanical behavior in presence of a higher CB content. In 100/0 and 70/30, although not in such a distinctive manner as observed in 100/0 (Figure S4a), a first force peak was detected, which reflects an initial rupture of the structure, although subsequently the force continued to increase with the distance travelled by the probe. Additionally, in either 100/0-Ch or 70/30-Ch (Figure S4b), the presence of other ingredients typical of chocolate, caused this first force peak to match the maximum force, and it was simultaneous with the usual “snap” of chocolate followed by an abrupt force drop. Besides, this induced the area under curve (AUC) to be lower in the 100/0-Ch and 70/30-Ch chocolates than in the single 100/0 and 70/30 blends. In contrast, 50/50, 30/70 and 0/100 blends, as well as their chocolate counterparts, presented force–time profiles characteristic of a malleable structure, in which force increases with the distance travelled by the probe, and peaks indicating the breakdown of the structure are not observed, nor does a final drop of the force that increased constantly with distance/time. By using the texture profile analysis, a hardness value equivalent to 67 N has been recently reported for CB [5].

Results show that the OG incorporation into the CB/OG blends and CB/OG-Ch counterparts very significantly affects their hardness (Table 7).

In general terms, the values of both textural parameters diminished progressively as the OG percentage increased, possibly due to a eutectic softening effect [49]. It is clear that higher levels of CB, and therefore of solid fat content in the continuous phase, result in firmer chocolates, as previously observed [52]. A stronger hardness has been also associated with a denser aggregation in the fat-crystal network [55]. In turn, Li and Liu [19] found that hardness of dark chocolate was significantly higher than that of chocolates containing different oleogels, which was associated with a higher SFA content in CB, as compared to that of oleogels, and as it was also observed in this research (Table 1).

### 2.5. Oil-Binding Capacity (OBC) of CB/OG Blends and CB/OG-Ch Counterparts

The OBC values corresponding to both matrices are presented in Table 8. The results showed that in both matrices, and either after 24 or 48 h, the OBC showed an inverse relationship with respect to the OG content, although the presence of sugar and cocoa powder in the dispersed phase decreased the differences observed. In addition, only the oil loss of the 30/70 and 0/100 blends and 30/70-Ch and 0/100-Ch counterparts were significantly higher than those of the rest of the samples. In turn, after 48 h the OBC is also higher than after 24 h. Additionally, in a similar OG, Patel et al. [25] reported that no signs of oiling-out were observed after 3 months of storage at 5 °C, also indicating a good storage stability for the OG. In this study, although the OG (0/100) exhibited the most important oil loss, the OBC of the 0/100-Ch counterpart was also very high, with 94.9 and 93.2% values at 24 and 48 h, respectively (Table 8).

This result indicated the efficiency of the foam-templated approach to obtain an OG used as a partial replacer for CB (maximum 70%), maintaining an appropriate oil binding property while significantly lowering the SFA in chocolate.

### 2.6. Sensory Analysis of CB/OG-Based Chocolates (CB/OG-Ch)

Table 9 and Figure S5 show the mean scores that the 32 untrained panelists gave to the four sensory attributes (appearance, smell, texture and taste), the overall acceptability and purchase intention of the five chocolates made with the different CB/OG blends.

It is possible to observe that the scores tend to be lower with the increasing percentage of CB replacement with the OG. However, only the scores for appearance, texture and flavor of the 30/70-Ch and 0/100-Ch chocolates were significantly lower than those of the 100/0-Ch control chocolate. In addition, only 0/100-Ch had a significantly lower overall acceptability and purchase intention as compared with the other chocolates. Additionally, it can be observed that the radar plots of 100/0-Ch, 70/30-Ch, 50/50-Ch and 30/70-Ch present the same shape, almost overlapping (Figure S5), and only the one corresponding to the chocolate made without CB is clearly different from the rest. This result reflects that although the decrease of CB fat in the CB/OG blends affects the physico-chemical properties and organoleptic characteristics of the chocolates formulated with them, it would be possible to replace up to 70% of CB by the OG in the formulation of the chocolates without affecting their acceptability and purchase intention. This would make it possible to benefit from the fact that 30/70-Ch is much healthier than 100/0-Ch since it contains a slightly lower amount of fat (Table S1) and only 47 and 40% of palmitic and stearic FA that are present in conventional chocolates (Table 1). However, and in line with previous studies [56], it is not possible to completely replace CB with the OG in the control chocolate without negatively affecting its sensory properties, especially its texture. Nonetheless, since the flavor of the 0/100-Ch chocolate was not excessively unpleasant (Table 9), this OG could be used in applications in other chocolate products with a softer texture and different from that of the chocolates in this study, such as filling creams, truffle-type products or pastries.

There are few studies aimed at improving the lipid profile of chocolates by replacing CB with alternatives (CBE) or oleogels which include a sensory analysis of the final products obtained. De Clercq et al. [7] used a triangular test to determine, without referring to a specific characteristic or attribute, whether there was any sensory difference between chocolates formulated only with CBE and a chocolate made only with CB. These authors observed that the panelists were not able to detect significant differences in the sensory properties of the chocolates, even when the percentage of CB replacement was 100%.

## 3. Conclusions

In this work, an OG was developed as a substitute for CB in chocolates, using the foam-templated approach for the first time, and employing HPMC (2% *w*/*w*) as a structuring agent for HORSO (98% *w*/*w*). CB presented a characteristic rheological behavior of a strong gel with a low melting point, while the OG exhibited a weak gel-like structure and high thermal stability; these differences are attributable to their distinct FA composition. In the CB/OG blends containing both ingredients, an increase in the proportion of the OG results in the weakening of the crystalline network of CB, as reflected by the rheological, thermal, textural parameters and oil retention capacity; parameters that were lower as the percentage of CB replacement increased. However, a relevant aspect at the technological level is that although the CB/OG-based chocolate (CB/OG-Ch) counterparts presented different structural characteristics among each other, these differences were much lower than those observed among the CB/OG blends, which is attributed to the effect of the presence of other ingredients used in the formulation of the chocolates. All CB/OG-based chocolates made with total or partial replacement of CB with the OG presented an improved lipid profile compared with the 100/0-Ch control chocolate. This improvement was evidenced by a reduction in the SFA content and an increase in MUFA and PUFA, proportional to the amount of CB replaced. However, although the sensory analysis showed that it would be possible to replace up to 70% of CB by the OG in the formulation of the chocolates without negatively affecting their sensory attributes, overall acceptability, and purchase intention; as regards technological properties, it would be advisable not to exceed a maximum substitution of 50%. It could be highly interesting to evaluate the viability of other oils of vegetable and marine origin rich in MUFA and PUFA in the development of this OG using the foam-templated approach, and to study their potential applicability not only in chocolate products, but also in the formulation of other foods that are rich in saturated fats (pâtés, spreadable products as margarine and cheese, filling creams, etc.).

## 4. Materials and Methods

### 4.1. Materials

The ingredients used to obtain the OG were distilled water, high oleic (80%) refined sunflower oil (HORSO) “Capicua” purchased from Coreysa (Sevilla, Spain) and one type of HPMC (METHOCEL™ F4M Food Grade) provided by The Dow Chemical Company (Bomlitz, Germany). Additional chemical specifications about the HPMC used were previously provided [6,31]. CB/OG blends were prepared with CB supplied by Barry Callebaut Iberica (Barcelona, Spain). The rest of the ingredients used to prepare the chocolate counterparts (CB/OG-Ch samples) were skimmed milk powder obtained from Capsa food (Asturias, Spain), cocoa powder “La Chocolatera” manufactured by Chocolates Valor S.A. (Alicante, Spain), fluid soy lecithin supplied by Manuel Riesgo S.A. (Madrid, Spain), and sifted white sugar from AB Azucarera Iberia SLU (Madrid, Spain).

### 4.2. Foam-Structured HPMC and OG Preparation

The HPMC template structure was prepared using an approach adapted from previous research [25,27,28,30] with slight modifications. HPMC was dissolved in distilled water to produce 1% (*w*/*w*) fully hydrated solutions. Each solution was foamed in 400 mL batches. Firstly, 99 g of hot distilled water (at 80 °C) were slowly mixed with 4 g of HPMC under slight agitation using a Bunsen (AGV-8) rod stirrer at low speed (300 rpm), and then the remaining water was cooled (to 1 °C) and added slowly with continuous stirring. The viscosity of the 1% fully hydrated HPMC solution was 230 ± 43.6 mPa s (measured at 20 °C and shear rate of 10 s^−1^). The hydrated HPMC was then aerated or foamed using a high dispersing unit IKA T25 basic (Ultraturrax^®^) at 13,500 rpm for 20 min. Overrun and foam stability of foamed HPMC were calculated in accordance with Patel et al. [25], being of 174 ± 14% and 90 ± 2.9%, respectively. The aqueous foam was frozen at −80 °C for 24 h, and then freeze-dried using a Beta 2–8 LDplus Version 1.76 lyophilizer (Martin Christ Gefriertrocknungsanlagen GmbH, Osterode, Germany). Complete water removal gave rise to the formation of a high-oil affinity porous cryogel or HPMC template, which was ground using a laboratory A320R1 grinder (Moulinex, Gruope Seb Ibérica, Barcelona, Spain) and mixed by hand with HORSO (98% *w*/*w*) to obtain samples containing 2% (*w*/*w*) freeze dried HPMC. The viscosity of HORSO was 55 mPa s (measured at 20 °C and shear rate of 1 s^−1^). The final HPMC-based OG was obtained by shearing the oil-sorbed freeze dried HPMC using the Ultraturrax^®^ at 11,000 rpm for 1 min, and left overnight at 4 °C until further use.

### 4.3. CB/OG Blend Preparation

The obtained OG was incorporated as a substitute for CB (100/0, control) in the formulation of the designed CB/OG blends at four different replacement levels (30, 50, 70 and 100%), thus designated as 70/30, 50/50, 30/70 and 0/100 based on the proportion of CB to the OG. 70/30, 50/50 and 30/70 blends were prepared by adding the corresponding amount of melted CB (50 °C for 60 min) to the adequate amount of warmed OG (at 40 °C), using a Bunsen (AGV-8) rod stirrer at 300 rpm. Each formulated CB/OG blend was then poured into plastic buckets, cooled and stored at 4 °C for 24 h until further use. In the case of the blends designated as 100/0 and 0/100, melted genuine CB and the pure OG, were directly molded and stored under identical conditions.

### 4.4. CB/OG-Based Chocolate (CB/OG-Ch) Preparation

The same four different CB/OG blends were incorporated into the formulation of chocolates as CB (100/0-Ch, control) replacers, and chocolate counterparts were designated as 70/30-Ch, 50/50-Ch, 30/70-Ch and 0/100-Ch. All chocolates were formulated with the corresponding CB/OG blend, sugar, milk powder, cocoa powder, and lecithin at 30, 32, 25, 12 and 1% percentages, respectively. Therefore, 100/0-Ch and 0/100-Ch contained solely CB and the OG at 30%, respectively, whereas 70/30-Ch, 50/50-Ch and 30/70-Ch contained both CB and OG ingredients at 21 and 9%, 15 and 15%, and 9 and 21%, respectively. Table S1 shows the theoretical nutritional composition information estimated for each formulated chocolate CB/OG-Ch, which was dependent on the added CB/OG blend. To make chocolates, firstly, each CB/OG combination was incubated at 50 °C for 60 min for melting, although the 0/100-Ch sample without CB did not melt and was able to retain its shape after this treatment. Then, the lecithin was dispersed in the corresponding CB/OG blend under agitation at low speed (200 rpm) for 5 min. Following, cocoa powder, milk powder and sugar, which were previously mixed by stirring at 40 rpm for 20 min, were slowly incorporated also under agitation until the total mixture was completely homogeneous. All chocolates were placed in silicon molds, allowed to cool, and stored at 4 °C for 24 h until further use.

### 4.5. Lipid Profile

Fatty acid (FA) contents of the five CB/OG-Ch chocolate formulations were determined by saponification and bimethylation as described by Alvarez et al. [6] using C13:0 as internal patron. Fatty acid methyl ester (FAME) was analyzed by gas chromatography on an Agilent gas chromatograph (Model 7820A, CA-USA) fitted with a GC-28 Agilent DB-23 capillary column (60 m × 250 µm × 0.25 μm), and a flame ionization detector was used. FAs were expressed as mg of fatty acid/g chocolate. Other nutritional factors, such as the atherogenic (AI) and thrombogenic (TI) indexes were also calculated [42].

### 4.6. Rheological Measurements

Rheological measurements of the CB/OG blends and the CB/OG-Ch chocolates were carried out with a rotational Kinexus pro rheometer (Malvern Instruments Ltd., UK) equipped with rSpace software and a high temperature cartridge in the lower plate for temperature control (resolution to 0.01 °C). A plate–plate measuring geometry PU40:PLS61X S3335SS, with a smooth upper plate of 40 mm of diameter and a serrated lower plate of 61 mm, was used (1.5-mm gap). Before measurements, all the samples were heated in a water bath at 50 °C for 60 min, and then transferred to the rheometer set at 20 °C.

#### 4.6.1. Dynamic Viscoelastic Properties

To determine the linear viscoelastic (LVE) range, amplitude sweep stress- or strain-controlled tests were run in both matrices at 20 °C and at 1 Hz with the shear stress or shear strain of the input signal varying from 2 to 200 Pa or from 0.01 to 100%, depending on the level of replacement of CB with the OG. Strain controlled tests were used for 0, 30 and 50% replacements of CB as the stress required to be registered out of the LVE range became higher with decreasing levels of CB replacement. Then, frequency sweeps were performed in both matrices at 20 °C between 10 and 0.1 Hz at chosen stress within the LVE rang. From frequency sweeps, the values of storage modulus (*G*’), loss modulus (*G*”) and loss tangent (tan *δ* = *G*”/*G*’) at 1 Hz, being the intermediate frequency of the frequency interval applied, were recorded. Additionally, in the CB/OG blends, temperature sweeps were carried out from 10 to 40 °C at a linear heating rate of 1 °C/min, and a frequency of 1 Hz within the LVE region.

#### 4.6.2. Flow Behavior

In both the CB/OG blends and the CB/OG-Ch counterparts, flow curves were obtained at 40 °C as recommended by ICA (2000) [57]. Firstly, a pre-shearing was applied at 5 s^−1^ for 15 min; subsequently, shear rate was increased stepwise from 2 to 50 s^−1^ for 5 min; then, shear rate was kept constant at 50 s^−1^ for 3 min; and finally, shear rate was decreased stepwise from 50 to 2 s^−1^ for 5 min. The Casson yield stress and Casson plastic viscosity values were calculated by fitting the data with the Casson model. In addition, the thixotropy of the samples was obtained by calculating the difference between the stress at 5 s^−1^ from the ramps up and down in agreement with other authors [19,48,53].

### 4.7. Thermal Parameters

A differential scanning calorimeter (TA Q1000, TA Instruments, New Castle, DE, USA) was employed to analyze the thermal behavior of both the CB/OG blends and the CB/OG-Ch samples. A 15–20 mg amount of each sample was placed in an aluminum pan and hermetically sealed. An empty pan was used as a reference. Samples were equilibrated at 5 °C for 10 min, and then heated from 5 to 80 °C at a constant rate of 5 °C/min. The melting onset, peak and completion temperatures (*T*_mo_, *T*_mp_ and *T*_mc_) and the enthalpy of melting were calculated from the area of the peak endotherm using the Universal Analysis 2000 software (v. 4.1D, TA Instruments, New Castle, DE, USA).

### 4.8. Texture Measurements

A TA.HDPlus Texture Analyzer (Stable Micro Systems, Ltd., Godalming, UK) provided with Texture Exponent software (version 6.1.16.0), and equipped with a 30 kg load cell was used for penetration tests of both the CB/OG blends and the CB/OG-Ch counterparts. The test was carried out at 20 °C using a polyoxymethylene cylindrical probe (P/10, 10 mm Ø) that penetrated the sample to a depth of 6 mm at a rate of 1 mm/s. The hardness of the samples was recorded as the force (N) and the area (N s) under the force–time curve at the defined penetration distance.

### 4.9. Oil-Binding Capacity

Oil-binding capacity (OBC) of the CB/OG blends and the CB/OG-Ch counterparts was measured by calculating the amount of oil expressed naturally after 24 and 48 h at room temperature, following the procedure previously described by other authors [6,30] with some modifications.

### 4.10. Sensory Analysis

Sensory evaluation was carried out in a standardized test room with individual booths. A total of 32 untrained panelists (84.4% women and 15.6% men, 23–61 years old), students and employees of the ICTAN-CSIC, took part of the sensory characterization of the CB/OG-Ch samples. Panelists received each chocolate randomly labeled with a three-digit code to enable identification and scored all the five compound chocolates in one session. Chocolates were served at room temperature, and water and bread were provided as palate cleansers. Each panelist scored the appearance (visual phase) and the smell (olfactory phase), and then the texture and the taste. They used a 9-cm unstructured scale (anchored from 1 “weak” to 9 “strong”) to score the intensity of the four attributes. Subsequently, each panelist was also asked to evaluate the overall acceptability and purchase intention. Overall acceptability was evaluated by using a 9-point hedonic scale ranging from 1 (“dislike extremely”) to 9 (“like extremely”), and purchase intention was evaluated with a 5-point scale ranging from 1 (“I would definitely not buy it”) to 5 (“I would definitely buy it”).

### 4.11. Statistical Analysis

All the measurements/analyses were repeated at least three times, with samples prepared on different days (two batches). Statistical analysis of all the data obtained for both the CB/OG blends and the CB/OG-Ch counterparts was performed using an analysis of variance (one-way ANOVA) to study the effect of the different levels of CB replacement with the OG. Significant differences between pairs of means were evaluated with the Tukey test, using a 95% confidence interval (*p* < 0.05). Analyses were carried out using IBM SPSS statistical for Windows, Version 25.0 (IBM Corp., Armonk, NY, USA).

## Figures and Tables

**Table 1:** Lipid profile corresponding to the different formulated CB/OG-based chocolates (CB/OG-Ch).

mg Fatty Acid/g Chocolate	100/0-Ch	70/30-Ch	50/50-Ch	30/70-Ch	0/100-Ch
Palmitic C16:0	72.4 ± 0.61 ^a^	60.7 ± 0.79 ^b^	46.2 ± 0.55 ^c^	34.0 ± 1.1 ^d^	16.8 ± 0.44 ^e^
Stearic C18:0	102 ± 0.91 ^a^	82.5 ± 1.3 ^b^	60.5 ± 0.74 ^c^	41.0 ± 1.3 ^d^	14.9 ± 0.37 ^e^
Arachidic C20:0	3.14 ± 0.022 ^a^	2.73 ± 0.063 ^b^	2.14 ± 0.030 ^c^	1.69 ± 0.057 ^d^	1.02 ± 0.033 ^e^
Behenic C22:0	0.614 ± 0.031 ^e^	1.42 ± 0.0058 ^d^	1.63 ± 0.010 ^c^	2.27 ± 0.092 ^b^	2.72 ± 0.078 ^a^
Other SFA	1.46	1.52	1.40	1.42	1.32
∑ SFA	179 ^a^	149 ^b^	112 ^c^	80.4 ^d^	36.8 ^e^
Palmitoleic C16:1n7	0.682 ± 0.0085 ^a^	0.643 ± 0.0047 ^b^	0.533 ± 0.0047 ^c^	0.491 ± 0.020 ^d^	0.382 ± 0.0045 ^e^
Vaccenic C18:1n7	1.03 ± 0.025 ^d^	1.52 ± 0.012 ^c^	1.57 ± 0.022 ^c^	1.94 ± 0.065 ^b^	2.17 ± 0.060 ^a^
Oleic C18:1n9	95.1 ± 0.95 ^d^	147 ± 1.4 ^c^	153 ± 1.4 ^c^	192 ± 6.6 ^b^	218 ± 6.5 ^a^
Eicosenoic C20:1n9	0.190 ± 0.014 ^d^	0.375 ± 0.0021 ^c^	0.420 ± 0.0053 ^c^	0.572 ± 0.011 ^b^	0.707 ± 0.034 ^a^
∑ MUFA	97.0 ^d^	150 ^c^	156 ^c^	195 ^b^	221 ^a^
Linoleic C18:2n6	12.1 ± 0.14 ^d^	19.4 ± 0.13 ^c^	20.4 ± 0.14 ^c^	26.1 ± 0.91 ^b^	29.8 ± 0.86 ^a^
Linolenic C18:3n3	0.919 ± 0.0072 ^a^	0.902 ± 0.015 ^a^	0.750 ± 0.0022 ^b^	0.720 ± 0.027 ^b^	0.598 ± 0.031 ^c^
Other PUFA	0.049 ± 0.0027	0.0759 ± 0.0022	0.0753 ± 0.0033	0.103 ± 0.0029	0.111 ± 0.0027
∑ PUFA	13.1 ^d^	20.4 ^c^	21.2 ^c^	26.9 ^b^	30.5 ^a^
Nutritional indexes					
∑PUFA/∑SFA	0.073 ^e^	0.137 ^d^	0.190 ^c^	0.335 ^b^	0.829 ^a^
AI	0.673 ± 0.0045 ^a^	0.365 ± 0.0045 ^b^	0.268 ± 0.0045 ^c^	0.158 ± 0.0045 ^d^	0.071 ± 0.0045 ^e^
TI	3.04 ± 0.0046 ^a^	1.64 ± 0.0083 ^b^	1.19 ± 0.0043 ^c^	0.667 ± 0.0012 ^d^	0.251 ± 0.0012 ^e^

Values are given as mean (*n* = 6) ± standard deviation. CB, cocoa butter; OG, oleogel obtained by the foam-templated approach. ^a–e^ For the same fatty acid, values followed by the same letter within each row indicate no significant differences (*p <* 0.05) according to Tukey’s test. SFA, saturated fatty acids; MUFA, monounsaturated fatty acids; PUFA, polyunsaturated fatty acids; AI, atherogenic index; TI, thrombogenic index.

**Table 2:** Rheological parameters (limit values of LVE range) obtained at 20 °C from amplitude sweeps for the different formulated CB/OG blends.

CB/OG Blend	*σ*_max_ (Pa)	*γ*_max_ (%)	G*_max_ (kPa)	tan *δ* (-)
100/0	3098 ± 36 ^a^	0.0315 ± 0.0010 ^b^	9862 ± 428 ^a^	0.0250 ± 0.0023 ^b^
70/30	2860 ± 148 ^b^	0.0359 ± 0.0027 ^b^	7996 ± 714 ^b^	0.0292 ± 0.00037 ^b^
50/50	1756 ± 94 ^c^	0.0310 ± 0.0016 ^b^	5689 ± 602 ^c^	0.0886 ± 0.028 ^a^
30/70	142 ± 0.00 ^d^	0.0166 ± 0.00087 ^c^	855 ± 45 ^d^	0.136 ± 0.019 ^a^
0/100	142 ± 0.00 ^d^	0.149 ± 0.0092 ^a^	95.5 ± 5.9 ^d^	0.0983 ± 0.037 ^a^

Mean value (*n* = 6) ± standard deviation. CB, cocoa butter; OG, oleogel obtained by the foam-templated approach. *σ*_max_, maximum shear stress amplitude; *γ*_max_, maximum shear strain amplitude. G*_max_, maximum complex modulus; tan *δ*, loss factor (= *G”*/*G’*). ^a–d^ Values followed by the same letter within each column indicate no significant differences (*p* < 0.05). according to Tukey’s test.

**Table 3:** Viscoelastic properties at 1 Hz and within the LVE range obtained at 20 °C from frequency sweeps for the different formulated CB/OG blends and CB/OG-based chocolate (CB/OG-Ch) counterparts.

Matrix	*G*’ (kPa)	*G*” (kPa)	tan *δ* (-)
CB/OG blend			
100/0	10,043 ± 752 ^a^	265 ± 24 ^a^	0.0263 ± 0.00049 ^d^
70/30	7299 ± 401 ^b^	264 ± 41 ^a^	0.0362 ± 0.0036 ^c,d^
50/50	5526 ± 152 ^c^	236 ± 28 ^a^	0.0427 ± 0.0053 ^c^
30/70	801 ± 73 ^d^	94.3 ± 5.4 ^b^	0.118 ± 0.0040 ^a^
0/100	121 ± 6.1 ^d^	8.88 ± 0.065 ^c^	0.0733 ± 0.0042 ^b^
CB/OG-Ch			
100/0-Ch	9898 ± 176 ^a^	237 ± 71 ^b^	0.0239 ± 0.0070 ^c^
70/30-Ch	10,307 ± 15 ^a^	389 ± 12 ^a^	0.0377 ± 0.0012 ^c^
50/50-Ch	9247 ± 372 ^b^	294 ± 61 ^a,b^	0.0317 ± 0.0063 ^c^
30/70-Ch	947 ± 108 ^c^	116 ± 16 ^c^	0.122 ± 0.0068 ^b^
0/100-Ch	239 ± 9.5 ^d^	35.4 ± 0.76 ^c^	0.148 ± 0.0066 ^a^

Mean value (*n* = 6) ± standard deviation. CB, cocoa butter; OG, oleogel obtained by the foam-templated approach. *G*’, elastic modulus; *G*”, viscous modulus; tan *δ*, loss factor (= *Gʺ*/*Gʹ*). ^a–d^ For the same matrix, values followed by the same letter within each column indicate no significant. differences (*p* < 0.05) according to Tukey’s test.

**Table 4:** Viscoelastic moduli at 1 Hz and at 10 and 30 °C, and crossover temperature from temperature sweeps for the different formulated CB/OG blends.

	10 °C		30 °C		Crossover Temperature (°C)
CB/OG Blend	*G*’ (kPa)	*G*” (kPa)	*G*’ (Pa)	*G*” (Pa)	
100/0	10,475 ± 45 ^a^	297 ± 18 ^a,b^	0.0145 ± 0.0044 ^b^	0.344 ± 0.071 ^b^	26.0 ± 0.0058 ^a^
70/30	7690 ± 1155 ^b^	252 ± 90 ^b^	0.0142 ± 0.0016 ^b^	0.640 ± 0.14 ^b^	23.0 ± 0.21 ^b^
50/50	9283 ± 670 ^a,b^	396 ± 13.3 ^a^	0.0253 ± 0.0043 ^b^	1.56 ± 0.078 ^b^	22.4 ± 0.51 ^b^
30/70	3795 ± 61.5 ^c^	223 ± 29.8 ^b^	0.144 ± 0.058 ^b^	1.87 ± 0.45 ^b^	22.2 ± 1.8 ^b^
0/100	38.5 ± 11.9 ^d^	2.94 ± 0.94 ^c^	28,817 ± 9469 ^a^	2559 ± 978 ^a^	-

Mean value (*n* = 6) ± standard deviation. CB, cocoa butter; OG, oleogel obtained by the foam-templated approach. *G*’, elastic modulus; *G*”, viscous modulus. ^a–d^ Values followed by the same letter within each column indicate no significant differences (*p* < 0.05) according to Tukey*’*s test.

**Table 5:** Casson yield stress, Casson plastic viscosity and thixotropy deduced at 40 °C from steady shear measurements for the different formulated CB/OG blends and CB/OG-based chocolate (CB/OG-Ch) counterparts.

Matrix	*σ*_CA_ (Pa)	*η*_CA_ (Pa s)	*R* ^2^	Thixotropy at 5 s^−1^ (Pa)
**CB/OG Blend**				
100/0	0.0000512 ± 0.0000025 ^c^	0.0303 ± 0.0012 ^c^	0.999 ± 0.00013	0.0073 ± 0.00030
70/30	0.999 ± 0.33 ^c^	0.561 ± 0.028 ^c^	0.945 ± 0.044	2.81 ± 2.3 ^b,c^
50/50	3.45 ± 0.45 ^c^	2.00 ± 0.16 ^b^	0.958 ± 0.026	9.75 ± 3.3 ^a,b^
30/70	10.4 ± 1.3 ^b^	2.58 ± 0.75 ^b^	0.997 ± 0.0027	2.73 ± 1.4 ^b,c^
0/100	20.3 ± 2.7 ^a^	3.97 ± 0.42 ^a^	0.968 ± 0.022	16.0 ± 6.1 ^a^
**CB/OG-Ch**				
100/0	4.53 ± 0.41 ^c^	0.857 ± 0.077 ^a^	0.960 ± 0.047	11.4 ± 1.1 ^b^
70/30	7.11 ± 0.15 ^b^	0.858 ± 0.093 ^a^	0.983 ± 0.0012	27.8 ± 6.3 ^a^
50/50	2.93 ± 0.24 ^d^	0.541 ± 0.083 ^b^	0.989 ± 0.0062	10.8 ± 1.3 ^b^
30/70	9.66 ± 0.81 ^a^	1.03 ± 0.11 ^a^	0.986 ± 0.0039	27.1 ± 1.9 ^a^
0/100	8.82 ± 0.35 ^a^	1.00 ± 0.056 ^a^	0.994 ± 0.0032	13.6 ± 1.7 ^b^

Mean value (*n* = 6) ± standard deviation. CB, cocoa butter; OG, oleogel obtained by the foam-templated approach. *σ*_CA_, Casson yield stress; *η*_CA_, Casson plastic viscosity. ^a–d^ For the same matrix, values followed by the same letter within each column indicate no significant differences (*p* < 0.05) according to Tukey’s test.

**Table 6:** Thermal parameters of the different formulated CB/OG blends and CB/OG-based chocolate (CB/OG-Ch) counterparts.

Matrix	T_mo_ (°C)	*T*_mp_ (°C)	*T*_mc_ (°C)	*ΔH*_m_ (J/g)
CB/OG Blend				
100/0	12.6 ± 0.65 ^a^	22.1 ± 0.064 ^b^	30.6 ± 1.1 ^a^	65.0 ± 2.7 ^a^
70/30	10.7 ± 0.14 ^b^	22.7 ± 0.085 ^a^	29.8 ± 0.13 ^a,b^	36.9 ± 1.5 ^b^
50/50	10.8 ± 0.19 ^b^	20.6 ± 0.057 ^c^	28.0 ± 0.085 ^b,c^	27.3 ± 1.7 ^c^
30/70	10.7 ± 0.30 ^b^	18.8 ± 0.18 ^d^	26.5 ± 0.47 ^c^	14.9 ± 0.21 ^d^
0/100	-	-	-	-
CB/OG-Ch				
100/0	12.9 ± 0.17 ^a^	22.8 ± 0.57 ^a^	31.7 ± 0.76 ^a,b^	19.6 ± 1.2 ^a^
70/30	12.5 ± 0.085 ^a^	24.0 ± 0.57 ^a^	32.0 ± 0.26 ^a^	14.6 ± 0.97 ^b^
50/50	12.8 ± 1.2 ^a^	22.7 ± 0.42 ^a^	30.3 ± 1.7 ^a,b^	7.58 ± 0.11 ^c^
30/70	11.9 ± 0.64 ^a^	21.7 ± 0.74 ^a^	28.1 ± 0.26 ^b^	6.02 ± 0.81 ^c^
0/100	-	-	-	-

Mean value (*n* = 6) ± standard deviation. CB, cocoa butter; OG, oleogel obtained by the foam-templated approach. *T*_mo_, *T*_mp_ and *T*_mc_, onset, peak and completion melting temperatures, respectively; *ΔH*_m_, melting enthalpy. ^a–d^ For the same matrix, values followed by the same letter within each column indicate no significantdifferences (*p* < 0.05) according to Tukey’s test.

**Table 7:** Textural penetration parameters obtained at 20 °C in the different formulated CB/OG blends and CB/OG-based chocolate (CB/OG-Ch) counterparts.

Matrix	Force at 6 mm (N)	AUC (N s)
CB/OG blend		
100/0	125 ± 8.5 ^a^	1198 ± 64 ^a^
70/30	98.2 ± 0.91 ^b^	954 ± 48 ^b^
50/50	17.0 ± 0.085 ^c^	127 ± 9.4 ^c^
30/70	10.6 ± 0.087 ^c,d^	83.5 ± 4.0 ^c^
0/100	0.132 ± 0.0064 ^d^	115 ± 2.7 ^c^
CB/OG-Ch		
100/0-Ch	189 ± 15 ^a^	575 ± 42 ^a^
70/30-Ch	123 ± 13 ^b^	520 ± 16 ^a^
50/50-Ch	43.6 ± 2.4 ^c^	337 ± 19 ^b^
30/70-Ch	20.2 ± 1.1 ^c,d^	142 ± 9.3 ^c^
0/100-Ch	3.07 ± 0.29 ^d^	15.1 ± 0.79 ^d^

Mean value (*n* = 6) ± standard deviation. CB, cocoa butter; OG, oleogel obtained by the foam-templated approach. AUC, area under force vs. time penetration curve. ^a–d^ For the same matrix, values followed by the same letter within each column. indicate no significant differences (*p* < 0.05) according to Tukey’s test.

**Table 8:** Oil-binding capacity (OBC) in the different formulated CB/OG blends and CB/OG-based chocolate (CB/OG-Ch) counterparts.

Matrix	OBC (%) at 24 h	OBC (%) at 48 h
CB/OG blend		
100/0	100 ± 0.00 ^a^	100 ± 0.00 ^a^
70/30	99.9 ± 0.015 ^a^	99.3 ± 0.56 ^a^
50/50	99.5 ± 0.071 ^a^	98.4 ± 0.58 ^a^
30/70	91.5 ± 0.73 ^b^	84.9 ± 0.63 ^b^
0/100	77.2 ± 1.8 ^c^	64.3 ± 1.65 ^c^
CB/OG-Ch		
100/0	100 ± 0.00 ^a^	100 ± 0.00 ^a^
70/30	100 ± 0.00 ^a^	100 ± 0.00 ^a^
50/50	100 ± 0.00 ^a^	100 ± 0.00 ^a^
30/70	99.0 ± 0.35 ^b^	98.1 ± 0.21 ^b^
0/100	94.9 ± 0.13 ^c^	93.2 ± 0.27 ^c^

Mean value (*n* = 6) ± standard deviation. CB, cocoa butter; OG, oleogel obtained by the foam-templated approach. ^a–c^ For the same matrix, values followed by the same letter within each column indicate no. significant differences (*p* < 0.05) according to Tukey’s test.

**Table 9:** Scores rated by the untrained panel on the sensory attributes, overall acceptability and purchase intention of the CB/OG-based chocolates (CB/OG-Ch) formulated with the different CB/OG blends.

CB/OG-Ch	Appearance	Odor	Texture	Taste	Global Acceptability	Purchase Intention
100/0-Ch	7.78 ± 1.1 ^a^	7.67 ± 1.3 ^a^	6.84 ± 1.7 ^a^	7.51 ± 1.3 ^a^	7.30 ± 1.3 ^a^	3.94 ± 1.0 ^a^
70/30-Ch	6.85 ± 1.5 ^a^	6.98 ± 1.6 ^a^	6.99 ± 1.5 ^a^	6.70 ± 1.8 ^a,b^	6.87 ± 1.4 ^a^	3.78 ± 1.1 ^a^
50/50-Ch	7.21 ± 1.0 ^a^	6.97 ± 1.2 ^a^	6.50 ± 1.9 ^a,b^	6.93 ± 1.8 ^a,b^	6.92 ± 1.7 ^a^	3.56 ± 1.2 ^a^
30/70-Ch	5.73 ± 1.6 ^b^	6.76 ± 1.5 ^a^	5.51 ± 2.1 ^b^	6.10 ± 1.9 ^b,c^	6.28 ± 1.8 ^a^	3.34 ± 1.3 ^a^
0/100-Ch	2.94 ± 1.8 ^c^	4.14 ± 2.0 ^b^	2.82 ± 1.8 ^c^	5.27 ± 2.1 ^c^	3.68 ± 2.1 ^b^	1.72 ± 1.0 ^b^

Mean value (*n* = 32) ± standard deviation. CB, cocoa butter; OG, oleogel obtained by foam-templated approach. ^a–c^ Values followed by the same letter within each column indicate no significant differences (*p* < 0.05) according to Tukey’s test.

## Data Availability

Data are contained within the article or Supplementary Materials.

## Supplementary Materials

The following are available online at https://www.mdpi.com/article/10.3390/gels7040220/s1, Figure S1: Complex modulus (*G**) as a function of the applied shear stress at 1 Hz and at 20 °C for the CB/OG-Ch chocolates. Shear stress ranged from 2 to 10,000 Pa depending on the CB/OG-Ch chocolate measured. Figure S2: Viscoelastic moduli (*G*’: filled symbols; *G*”: open symbols) as a function of the frequency at 20 °C. Stress stress ranged from 100 to 1000 Pa within the LVE range depending on the blend or chocolate measured: (**a**) for the CB/OG blends; (**b**) for the CB/OG-Ch chocolates. Figure S3: Thermograms obtained by heating from 5 to 80 °C at a constant rate of 5 °C/min: (**a**) for the CB/OG blends; (**b**) for the CB/OG-Ch counterparts. Figure S4: Force–time curves at 20 °C: (**a**) for the CB/OG blends; (**b**) for the CB/OG-Ch chocolates. Figure S5: Radar plots representing perceived sensory attributes and overall acceptability of formulated CB/OG-Ch chocolates. Table S1: Theoretical nutritional composition of formulated CB/OG-Ch chocolates estimated according to the nutritional information shown on the packaging of each ingredient used in the manufacturing of the chocolates along with the Spanish Food Composition Database (BEDCA).
