# Supplementary material for: Development of Chocolates with Improved Lipid Profile by Replacing Cocoa Butter with an Oleogel
Source: Gels. 2021 Nov 18;7(4):220. doi: 10.3390/gels7040220 (PMC8628694; doi:10.3390/gels7040220)
Supplement: Supplementary file 1 [file gels-07-00220-s001.zip › gels-1387706-supplementary.pdf]

Table S1. Theoretical nutritional composition of formulated CB/OG-Ch chocolates estimated according to the nutritional information shown on the packaging of each ingredient used in the manufacturing of the chocolates along with the Spanish Food Composition Database (BEDCA).

Cocoa powder (g)						Skimmed milk powder (g)						Lecithin (g)	Sugar (g)	CB/OG blend (g)		Nutritional composition of chocolates	
12						25						1	32	30		**F (%)**	**33.17**
F (%)	C (%)	W (%)	M (%)	P (%)	Fi (%)	F (%)	C (%)	W (%)	M (%)	S (%)	P (%)	F (%)	C (%)	F (%)	C (%)	**C (%)**	**47.58**
16	16.3	1.5	3.8	F (%)	31.7	1	54.5	3.5	3.9	1.3	32.5	100	100	100	0	P (%)	11.19
1.92	1.92	1.96	0.18	3.06	3.80	0.25	13.63	0.88	0.98	0.33	8.13	1.00	32.00	30.00	0.00	Fi (%)	3.80
																S (%)	0.33
**Composition of 100/0-Ch prepared with 100% CB (100/0 blend)**																W (%)	1.06
																M (%)	1.43
																**Total**	99
12						25						1	32	30		**F (%)**	**32.99**
F (%)	C (%)	W (%)	M (%)	P (%)	Fi (%)	F (%)	C (%)	W (%)	M (%)	S (%)	P (%)	F (%)	C (%)	F (%)	C (%)	**C (%)**	**47.76**
16	16.3	1.5	3.8	25.5	31.7	1	54.5	3.5	3.9	1.3	32.5	100	100	99.4	0.6	P (%)	11.19
1.92	1.96	0.18	0.46	3.06	3.80	0.25	13.63	0.88	0.98	0.33	8.13	1.00	32.00	29.82	0.18	Fi (%)	3.80
																S (%)	0.33
**Composition of 70/30-Ch prepared with 70% CB and 30% OG (70/30 blend)**																W (%)	1.06
																M (%)	1.43
																**Total**	99
12						25						1	32	30		**F (%)**	**32.87**
F (%)	C (%)	W (%)	M (%)	P (%)	Fi (%)	F (%)	C (%)	W (%)	M (%)	S (%)	P (%)	F (%)	C (%)	F (%)	C (%)	**C (%)**	**47.88**
16	16.3	1.5	3.8	25.5	31.7	1	54.5	3.5	3.9	1.3	32.5	100	100	99	1	P (%)	11.19
1.92	1.96	0.18	0.46	3.06	3.80	0.25	13.63	0.88	0.98	0.33	8.13	1.00	32.00	29.70	0.30	Fi (%)	3.80
																S (%)	0.33
**Composition of 50/50-Ch prepared with 50% CB and 50% OG (50/50 blend)**																W (%)	1.06
																M (%)	1.43
																**Total**	99
12						25						1	32	30		**F (%)**	**32.75**
F (%)	C (%)	W (%)	M (%)	P (%)	Fi (%)	F (%)	C (%)	W (%)	M (%)	S (%)	P (%)	F (%)	C (%)	F (%)	C (%)	**C (%)**	**48.00**
16	16.3	1.5	3.8	25.5	31.7	1	54.5	3.5	3.9	1.3	32.5	100	100	98.6	1.4	P (%)	11.19
1.92	1.96	0.18	0.46	3.06	3.80	0.25	13.63	0.88	0.98	0.33	8.13	1.00	32.00	29.58	0.42	Fi (%)	3.80
																S (%)	0.33
**Composition of 30/70-Ch prepared with 30% CB and 70% OG (30/70 blend)**																W (%)	1.06
																M (%)	1.43
																**Total**	99
12						25						1	32	30		**F (%)**	**32.57**
F (%)	C (%)	W (%)	M (%)	P (%)	Fi (%)	F (%)	C (%)	W (%)	M (%)	S (%)	P (%)	F (%)	C (%)	F (%)	C (%)	**C (%)**	**48.18**
16	16.3	1.5	3.8	25.5	31.7	1	54.5	3.5	3.9	1.3	32.5	100	100	98	2	P (%)	11.19
1.92	1.96	0.18	0.46	3.06	3.80	0.25	13.63	0.88	0.98	0.33	8.13	1.00	32.00	29.40	0.60	Fi (%)	3.80
																S (%)	0.33
**Composition of 0/100-Ch prepared with 100% OG (0/100 blend)**																W (%)	1.06
																M (%)	1.43
																**Total**	99

F, fat; C, carbohydrate; W, water; M, minerals; P, protein; Fi, fiber; S, salt; Database BEDCA (https://www.bedca.net/bdpub/index_en.php).

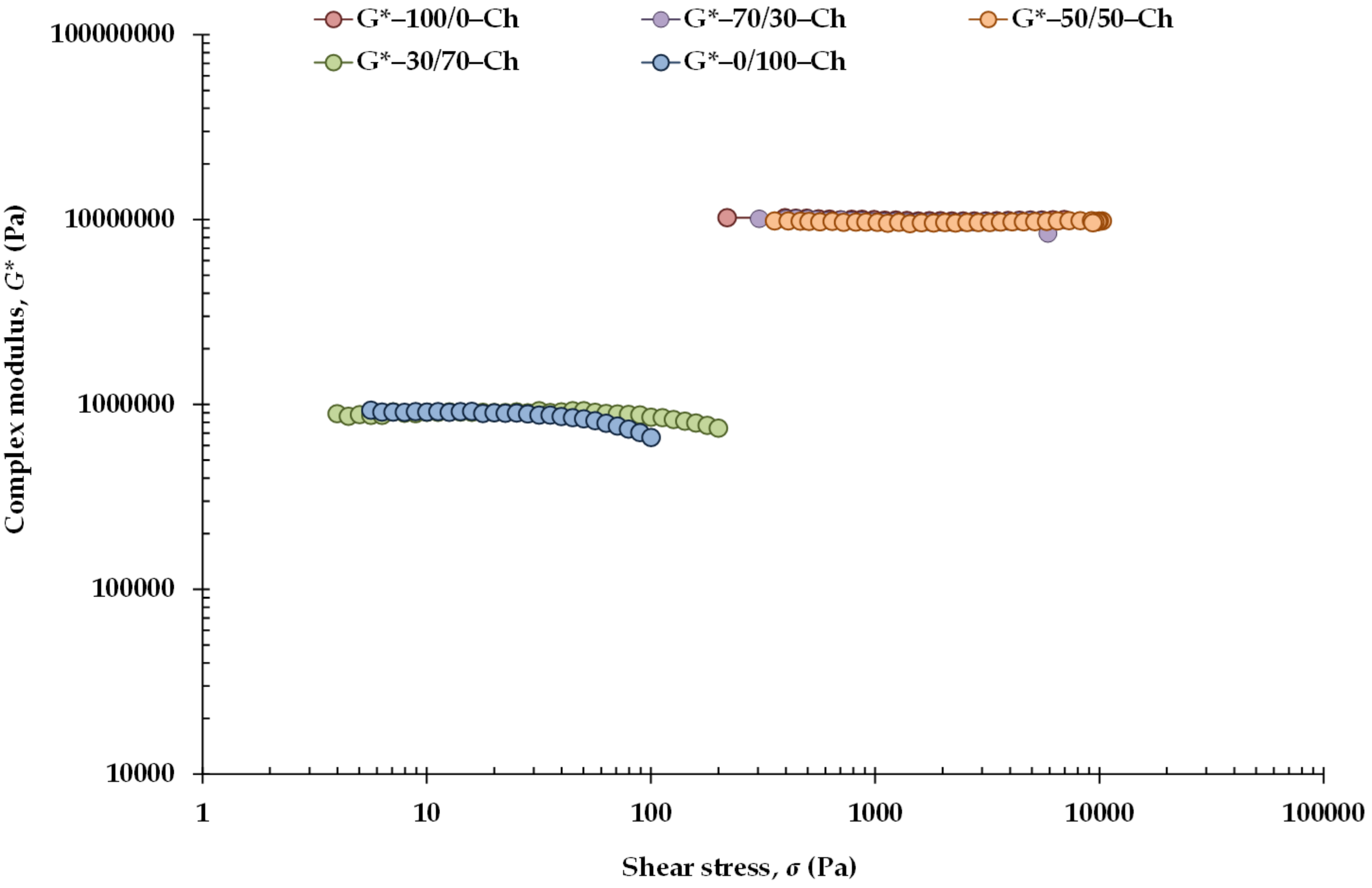

Figure S1. Complex modulus (*G**) as a function of the applied shear stress at 1 Hz and at 20 °C for the CB/OG-Ch chocolates. Shear stress ranged from 2 to 10000 Pa depending on the CB/OG-Ch chocolate measured.

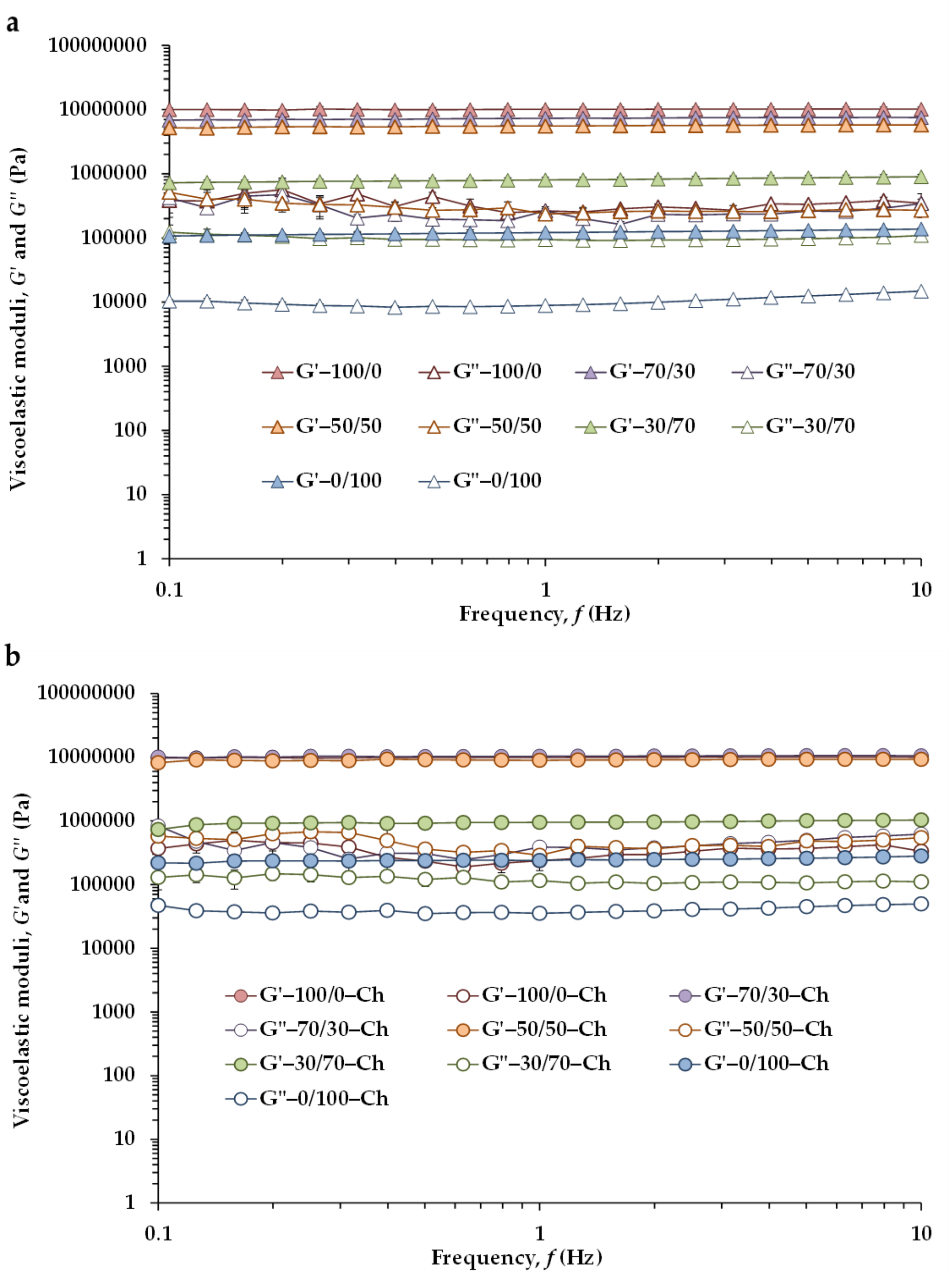

Figure S2. Viscoelastic moduli (*G*': filled symbols; *G*'': open symbols) as a function of the frequency at 20 °C. Shear stress ranged from 100 to 1000 Pa within the LVE range depending on the blend or chocolate measured: (**a**) for the CB/OG blends; (**b**) for the CB/OG-Ch chocolates.

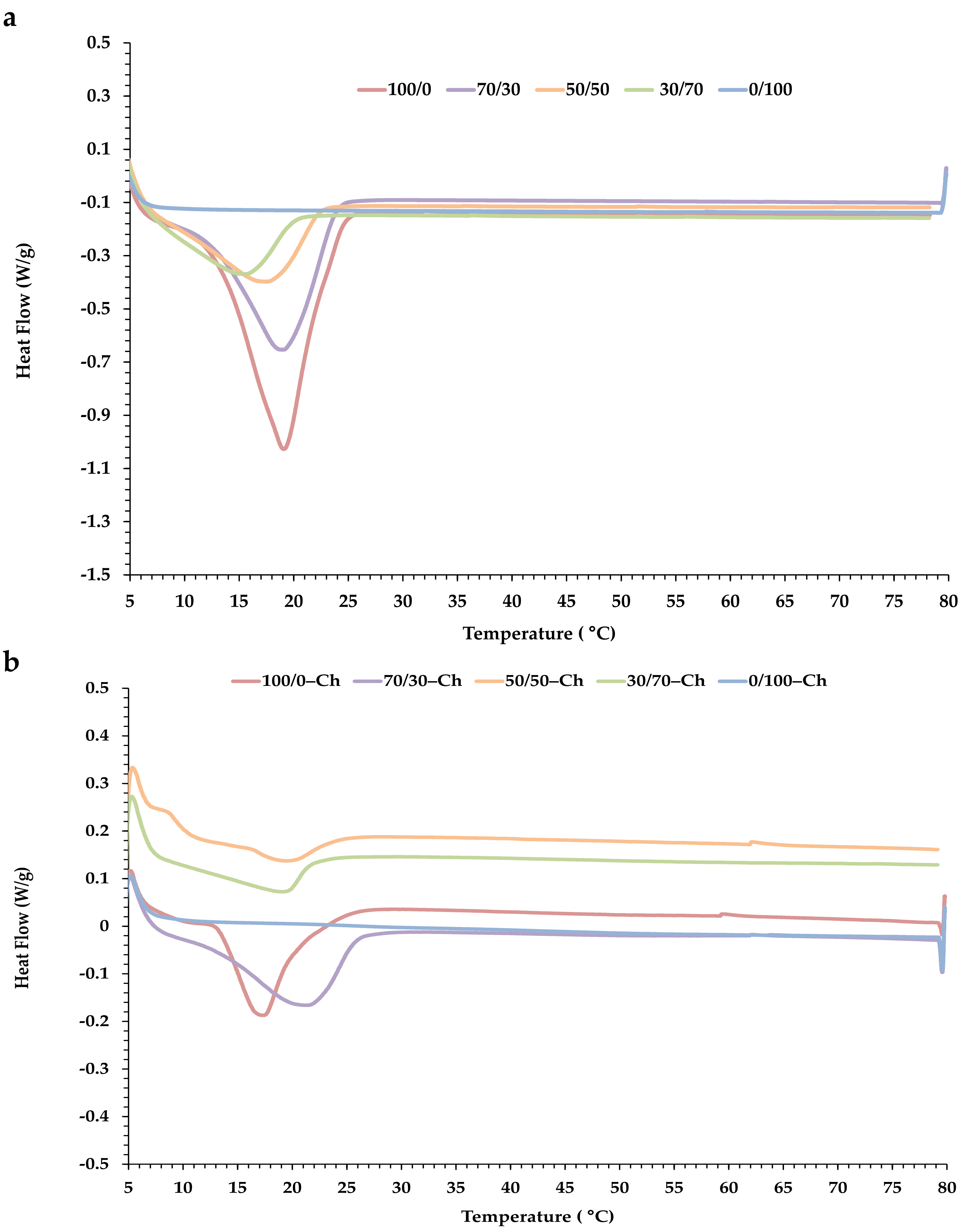

Figure S3. Thermograms obtained by heating from 5 to 80 °C at a constant rate of 5 °C/min: (**a**) for the CB/OG blends; (**b**) for the CB/OG-Ch counterparts.

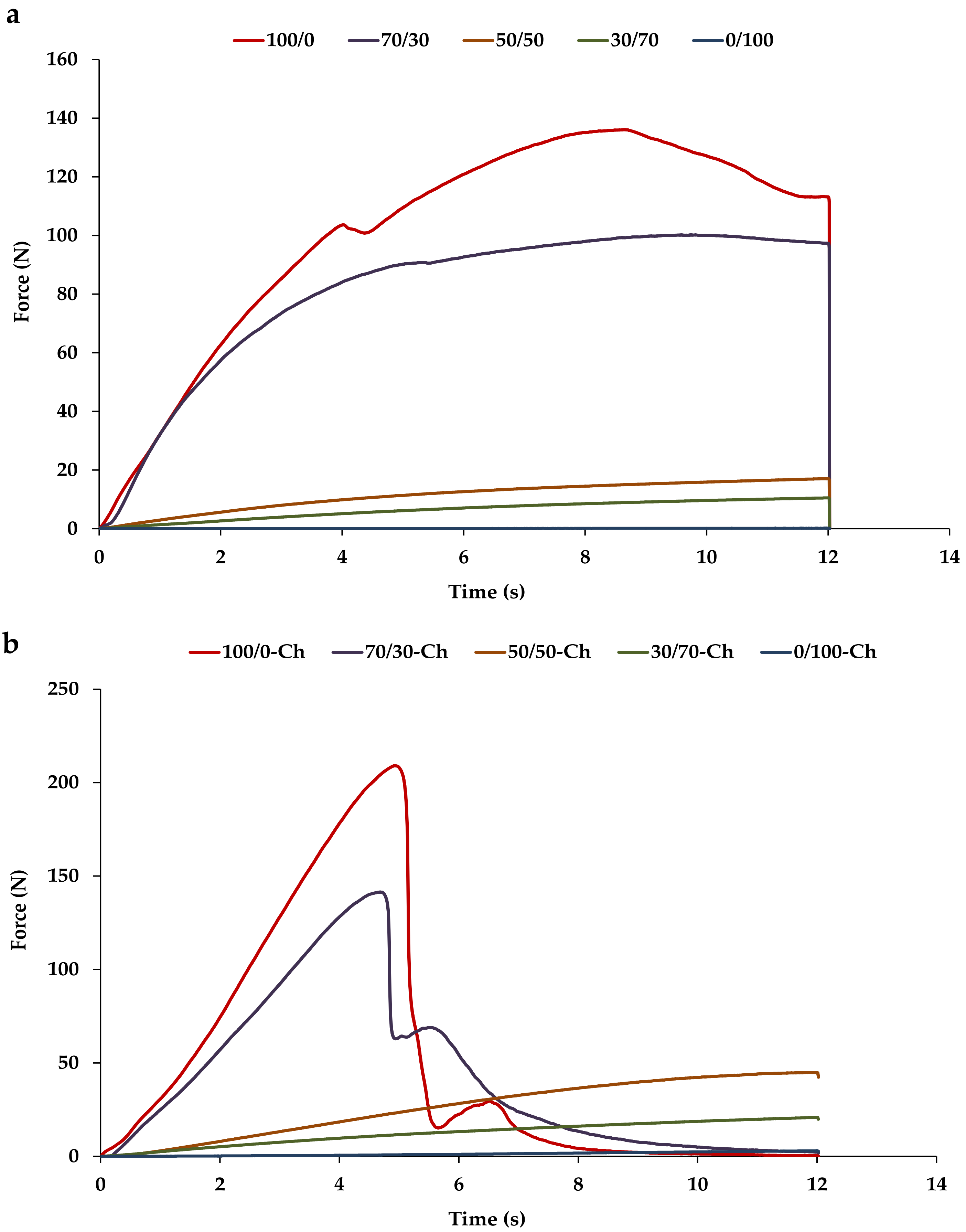

Figure S4. Force-time curves at 20 °C: (**a**) for the CB/OG blends; (**b**) for the CB/OG-Ch chocolates.

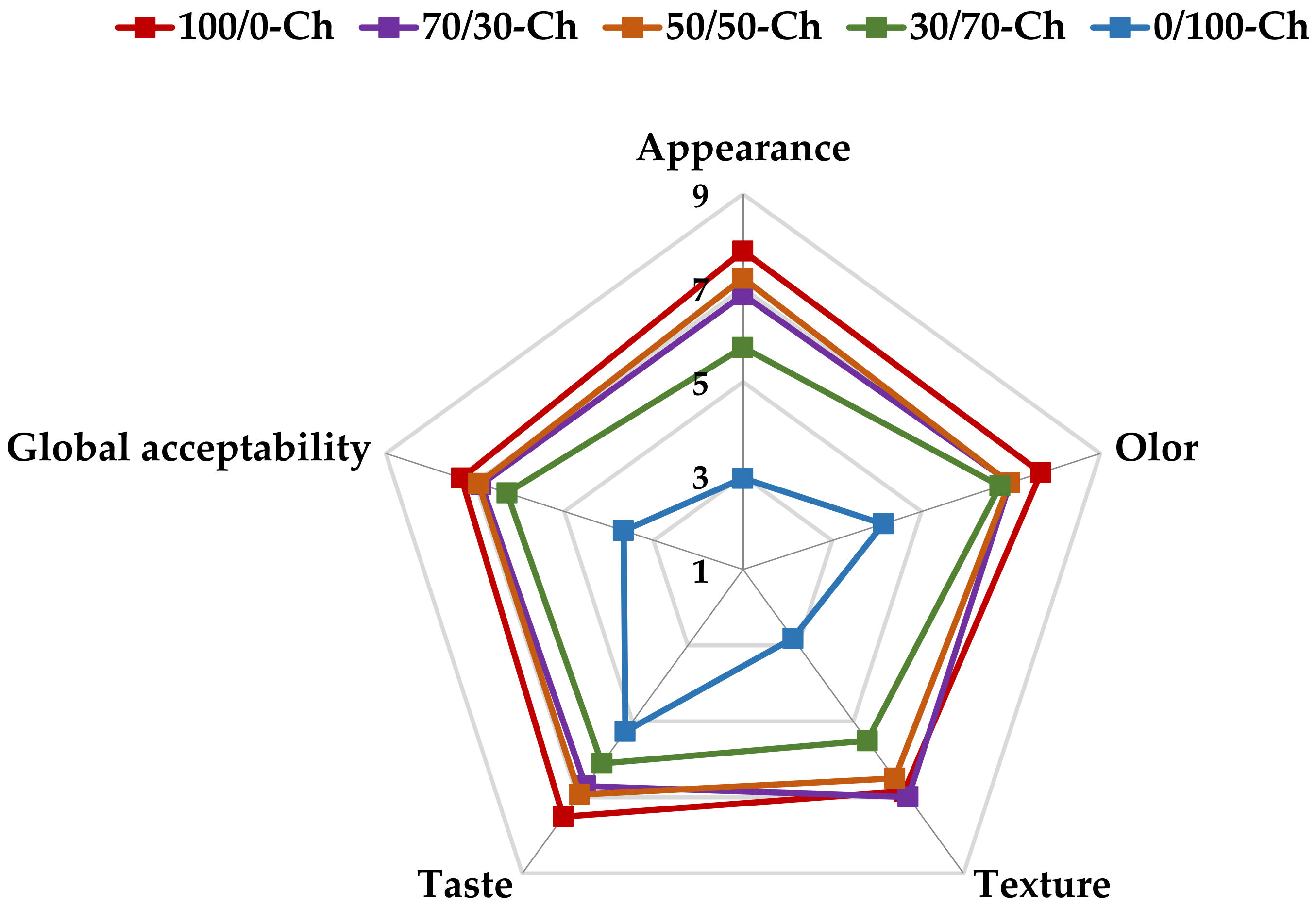

Figure S5. Radar plots representing perceived sensory attributes and global acceptability for the formulated CB/OG-Ch chocolates.
